# What you know can influence what you are going to know (especially for older adults)

**DOI:** 10.3758/s13423-014-0672-8

**Published:** 2014-06-12

**Authors:** Stephen P. Badham, Elizabeth A. Maylor

**Affiliations:** Department of Psychology, University of Warwick, Coventry, CV4 7AL UK

**Keywords:** Aging and memory, Recognition, Schemas, Face-name associations

## Abstract

Stimuli related to an individual’s knowledge/experience are often more memorable than abstract stimuli, particularly for older adults. This has been found when material that is congruent with knowledge is contrasted with material that is incongruent with knowledge, but there is little research on a possible graded effect of congruency. The present study manipulated the degree of congruency of study material with participants’ knowledge. Young and older participants associated two famous names to nonfamous faces, where the similarity between the nonfamous faces and the real famous individuals varied. These associations were incrementally easier to remember as the name–face combinations became more congruent with prior knowledge, demonstrating a graded congruency effect, as opposed to an effect based simply on the presence or absence of associations to prior knowledge. Older adults tended to show greater susceptibility to the effect than young adults, with a significant age difference for extreme stimuli, in line with previous literature showing that schematic support in memory tasks particularly benefits older adults.

Memory for a given stimulus can vary depending on an individual’s knowledge and experience with the to-be-remembered material. Chase and Simon (1973) demonstrated that expert chess players were better able to memorize arrangements of pieces in chess games than were novice chess players. Similarly, music experts have been shown to have better memory for passages of text about music than do nonmusic experts (Arbuckle, Vanderleck, Harsany, & Lapidus, 1990), architects have been shown to have better visuospatial working memory than do nonarchitects (Cavallini, Cornoldi, & Vecchi, 2009), and pilots have been shown to have superior memory for aviation-related material than do nonpilots (Morrow, Menard, Stine-Morrow, Teller, & Bryant, 2001). On a more general basis, it has also been shown that vocabulary level is positively correlated with word memory ability (see, e.g., Hultsch, Hertzog, & Dixon, 1990).

The above studies are based on individual differences, but there are also many circumstances in which material that is congruent with knowledge/experience is more easily remembered than material that is incongruent with knowledge/experience. For example, this occurs with recall of words and nonwords (Hulme, Maughan, & Brown, 1991), recognition of possible and impossible line drawings of objects (Schacter, Cooper, & Valdiserri, 1992), cued recall of related and unrelated word pairs (Badham, Estes, & Maylor, 2012), and cued recall of related and unrelated picture pairs (Sharps & Antonelli, 1997). It should also be noted that there are studies showing better memory for novel stimuli compared with familiar stimuli (e.g., the bizarreness effect, Gounden & Nicolas, 2012; McDaniel, Einstein, DeLosh, May, & Brady, 1995). However, novel/distinctive material is generally only more memorable when it is contrasted with familiar material within a given memory set (Hunt, 1995), and blocked novel stimuli infrequently show special mnemonic properties (Schmidt, 1991).

The notion that knowledge/experience can support memory processes has been widely explored in the context of cognitive aging. A variety of studies have shown that knowledge/experience can provide schematic support that disproportionately benefits older adults’ memory relative to that of young adults. Early studies using paired-associates tasks showed that age deficits in memory are alleviated with strongly associated words, relative to weakly associated words (Canestrari, 1966; Kausler & Lair, 1966). More recently, age deficits in memory for associations between words have been alleviated by semantic relations between to-be-remembered words (Badham et al., 2012; Naveh-Benjamin, Hussain, Guez, & Bar-On, 2003). This pattern of results extends beyond word pairs. Smith, Park, Earles, Shaw, and Whiting (1998) tested young and older adults’ memory for associations between pairs of related and unrelated pictures. Age deficits in memory were reduced when the pictures were related to each other, compared with when they were unrelated. Castel (2005) tested young and older adults’ memory for fictional prices of items that were either realistic (e.g., broccoli $1.89) or unrealistic (e.g., pickles $17.89). Age interacted with price type such that there was an age-related memory deficit for the unrealistic prices but not for the realistic prices. Similarly, McGillivray and Castel (2010) found age deficits in memory for unrealistic ages of faces but not for realistic ages of faces.

Although there is evidence for disproportionate schematic support of older adults’ memory, some studies show age invariance in the support of memory by schematic knowledge. Arbuckle et al. (1990) found that young and older music experts had superior memory for music passages compared with passages about dogs, whereas young and older nonmusic experts did not. Expertise benefitted the two age groups to the same extent. Similar results were found with young and older individuals who varied in cooking expertise and who memorized passages related or unrelated to cooking (Miller, 2003). Light and Anderson (1983) tested young and older adults’ memory for scripts describing typical and atypical actions, but age deficits in memory were similar across script types. These conflicting results indicate that it is important to develop further insight into how schematic support benefits memory in young and older adults.

The majority of studies investigating schematic support of memory have used highly contrasting material that is either consistent or inconsistent with participants’ knowledge/experience. One factor that has been overlooked is the effect of incremental changes in the consistency of stimulus material with an individual’s knowledge. The present article attempts to clarify whether degree of congruency with knowledge is important, as opposed to simply the presence or absence of prior representations of a stimulus. To our knowledge, only one paradigm has previously been used to examine continuous effects of prior knowledge on memory. Hemmer and Steyvers (2009) tested young adults’ memory for the presentation size of images of fruits and vegetables on a computer screen. They found that when participants reconstructed the sizes of images, they were biased toward the real size of the food (and its size within the category). For example, if a pineapple and a raspberry were displayed at the size of a pear, the raspberry would be remembered as smaller than it was displayed and the pineapple would be remembered as larger than it was displayed (for similar results, see Heussen, Poirier, Hampton, & Aldrovandi, 2011). Thus, only a small body of evidence exists of a graded benefit to memory on the basis of degree of congruency with existing knowledge.

The present study adopted a novel paradigm to assess how incremental changes in the congruency of a stimulus with prior knowledge would influence the memorability of that stimulus. Furthermore, the paradigm was based on associative memory, where older adults have consistently been shown to have particular deficits relative to young adults (Naveh-Benjamin, 2000; Old & Naveh-Benjamin, 2008). It is hypothesized that prior knowledge/schematic support will disproportionately benefit older adults’ associative memory relative to that of young adults. In brief, young and older participants were required to associate two famous names with a range of nonfamous faces that varied in their similarity to the famous individuals. We predicted that more similar name–face combinations would be remembered better than less similar name–face combinations, especially for older adults.

## Method

### Participants

Forty-eight young adults (33 female, ages 18–27 years, *M* = 20.0, *SD* = 1.9) and 48 older adults (33 female, ages 64–86 years, *M* = 73.1, *SD* = 5.4) took part in the experiment.^1^ Young and older adults had similar years of education, *M* = 14.6, *SD* = 1.6, and *M* = 15.0, *SD* = 2.8, respectively (*t* < 1). Young participants were either recruited from the university and compensated a small amount, or they participated without compensation as part of demonstration sessions at open days for the Department of Psychology. Older adults were recruited from the local community and were given compensation toward their travel expenses.

### Materials

Stimuli were 16 faces presented on a computer screen above one of two names. Two highly famous individuals’ names were used: Prince William (PW) and George Bush (GB). The faces were all nonfamous individuals, but two faces were lookalikes for PW and two were lookalikes for GB (lookalike images were taken from the Internet). The remaining faces were taken from an online database (Psychological Image Collection at Stirling; http://pics.psych.stir.ac.uk/). The faces were of white males who varied in their similarity to PW and GB.

The two pairs of lookalike faces were always presented with one of each name. That is, for the two PW lookalikes, one would be randomly presented with the name PW and the other would be presented with the name GB (and similarly for the GB lookalikes). For each participant, the remaining 12 faces were randomly named either PW or GB under the constraint that each name was used exactly six times. The order of presentation of faces was entirely random for each participant.

An independent group of 26 individuals (18 female, ages 23–78 years, *M* = 46.5, *SD* = 22.0, and with *M* = 17.5, *SD* = 1.5 years of education) ranked the faces in terms of their similarity to PW and GB. On a paper-based questionnaire, they were shown actual pictures of PW and GB and then were asked to rank the 16 nonfamous faces on the following basis: “Please rank the following 16 faces to establish if they are more similar to Prince William or to George Bush. Place a 1 to the right of the face most similar to Prince William and a 16 to the right of the face most similar to GB, so that each of the numbers 1–16 is used once.” The rankings were averaged together for each face, to establish their similarity to PW/GB. The lookalike faces appeared in the predicted positions, with the PW lookalikes falling in positions 1 and 2 and the GB lookalikes falling in positions 15 and 16. Shows the faces in rank order. For all 16 faces, the mean pairwise rater correlation was .78, with a 90 % confidence interval of .77–.79. For just the 12 non-lookalike faces, the mean pairwise rater correlation was .50, with a 90 % confidence interval of .47–.52.

### Procedure

After we established that each participant was familiar with PW and GB, participants viewed the 16 nonfamous faces with the name “Prince William” or “George Bush” displayed below them. Each face (and name) was shown for 3 s and a blank white screen was shown for 1 s before each stimulus. Participants were asked to remember which name was displayed with each face. Following encoding, there was a 30-s delay during which participants were required to respond “true” or “false” to the correctness of various simple numerical equations (e.g., 5 × 3 = 15: true). After this delay, a two-alternative forced-choice recognition memory test took place. Participants were shown each face, one at a time, in the center of the screen. Below the face, the names “Prince William” and “George Bush” were displayed on the left and right of the screen (counterbalanced across participants). Participants pressed left and right keys (“F” and “J”) to indicate which name they remembered being shown with the face in the encoding phase. Participants were informed of the test structure before they began the study phase.

## Results

**Fig. 1:**
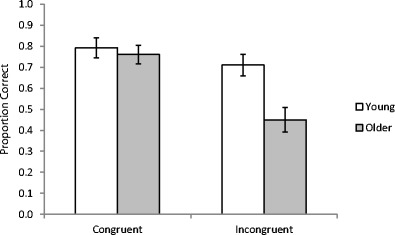
Memory accuracy for young and older adults for names congruent or incongruent with lookalikes’ appearances. Error bars are ±1*SE*

Initially, performance was assessed for the lookalike faces. The proportion of correct responses for remembering the name associated to each lookalike face was calculated. These responses were then categorized as congruent (e.g., remembering that the name PW was originally displayed with a lookalike for PW) or incongruent (e.g., remembering that the name GB was originally displayed with a lookalike for PW). A 2 (Age: young, older) × 2 (Congruency: congruent, incongruent) repeated-measures analysis of variance (ANOVA) was conducted on the accuracy data (see Fig. 1 for means). There was a main effect of age [*F*(1, 94) = 11.35, *MSE* = 0.10, *p* < .01, η_p_^2^ = .11], with young adults performing better than older adults. There was a main effect of congruency [*F*(1, 94) = 12.20, *MSE* = 0.15, *p* < .001, η_p_^2^ = .12], with more accurate memory for congruent than for incongruent stimuli. There was also a significant interaction between age and congruency [*F*(1, 94) = 4.08, *MSE* = 0.15, *p* < .05, η_p_^2^ = .04], with older adults showing a larger difference in accuracy between congruent and incongruent stimuli than did young adults. Age differences were absent for congruent name–face combinations (*t* < 1) and present for incongruent name–face combinations [*t*(94) = 3.36, *p* < .01].

**Fig. 2:**
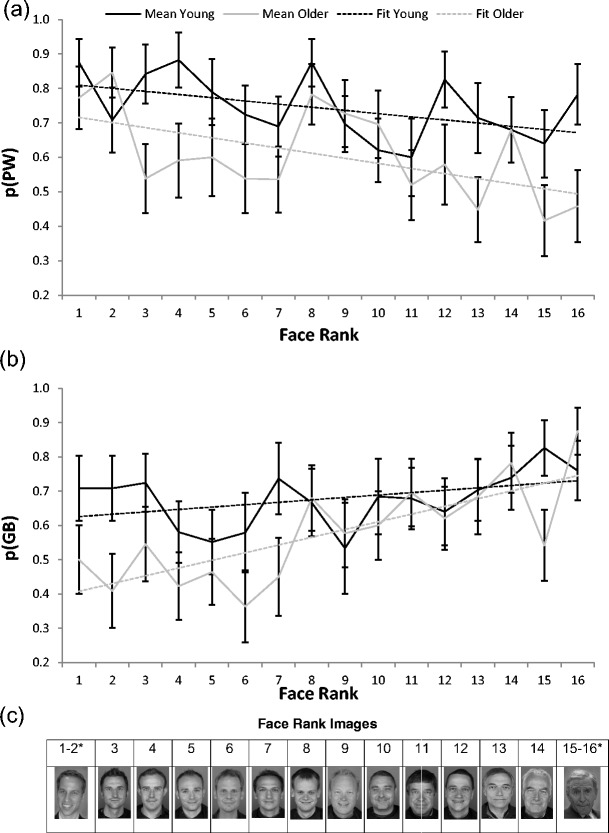
Performance for young and older adults remembering if the names Prince William (PW) or George Bush (GB) were displayed with each face, and the model fit to those data. Error bars are ±1*SE*. **a** Probability of correctly recalling that the name PW was shown with each face. **b** Probability of correctly recalling that the name GB was shown with each face. **c** Face stimuli ranked from 1 (most similar to PW) to 16 (most similar to GB). *Only one of each pair of lookalikes is presented as an example

In order to assess the effect of congruency across the whole data, lines of best fit (slopes and intercepts: *y* = *mx* + *b*) were estimated for recognition accuracy data after arranging the 16 faces in rank order of their similarity to PW/GB. Along the *x*-axis, the face ranked most similar to PW was in Position 1, with the rank increasing up to Position 16 for the face ranked most similar to GB. Two sets of lines of best fits were calculated, one with the *y*-axis as the probability of recognizing the name for each face when labeled “PW” at study, and another with the *y*-axis as the probability of recognizing the name for each face when labeled “GB” at study (see Fig. 2a and b). Maximum likelihood estimation was used to estimate the slopes and intercepts of the lines of best fit. In a general model, separate sets of lines were fit for young and older adults’ data, giving a total of four slopes and four intercepts. The general model was compared with three restricted models:

1. The first restricted model had the four slopes fixed at zero (i.e., testing if the slopes improved the fit to the data). A likelihood-ratio test confirmed that the general model fit significantly better than the restricted model after taking into account the extra degrees of freedom in the general model [*χ* ^2^(4) = 29.60, *p* < .001]. This indicated that there was an effect of congruency in the data, with slopes showing better memory for more congruent name–face combinations than for less congruent name–face combinations.
2. The second restricted model had lines of best fit that were not allowed to be different for young and older adults (i.e., testing if the two age groups responded differently to the stimuli). A likelihood-ratio test confirmed that the general model also fit significantly better than the second restricted model [*χ* ^2^(4) = 29.20, *p* < .001], demonstrating that young and older adults were responding differently to the task. This age difference may have been due to general age deficits in memory, and was investigated further in the third restricted model.
3. The third restricted model had slopes fixed as the same for young and older adults but the intercepts were allowed to vary for each age group (i.e., to test if young and older adults responded differently to congruency). The general model fit marginally better than the third restricted model [*χ* ^2^(2) = 4.99, *p* = .08]. The direction of this trend was in line with our predictions—namely, that older adults, with data leading to steeper slopes than young adults, were more affected by congruency.

It is possible that congruency effects were driven by responses to the lookalike faces, which represented the most extreme stimuli. To test for this, the general and restricted models above were fit to the data for non-lookalike faces only (face ranks 3–14 inclusive in Fig. 2c). The general model fit the data better than the first restricted model [*χ* ^2^(4) = 14.82, *p* < .01], indicating that congruency was still important for nonextreme stimuli and that knowledge/experience was influencing memory performance. The general model fit better than the second restricted model [*χ* ^2^(4) = 21.72, *p* < .001], indicating age differences in the data. The general model was also a marginally better fit than the third restricted model [*χ* ^2^(2) = 4.98, *p* = .08], indicating that older adults were somewhat more influenced by congruency than were young adults.

Finally, to investigate the marginal age differences in congruency effects, the general model was compared with the first restricted model separately for young and older adults to establish if slopes improved the fit for both age groups. With the data for all 16 of the faces, the effect of congruency was marginal for young adults [*χ* ^2^(2) = 5.08, *p* = .08], but highly significant for older adults [*χ* ^2^(2) = 24.52, *p* < .001]. With the data for the non-lookalike faces only, there was a weak congruency trend for young adults [*χ* ^2^(2) = 3.77, *p* = .15] and a significant effect of congruency for older adults [*χ* ^2^(2) = 11.06, *p* < .01]. This also suggests that the effect of congruency was stronger in older adults than in young adults.

## Discussion

The present data showed that material more congruent with knowledge/experience can be more easily memorized than material less congruent with knowledge/experience. This effect was more pronounced for older adults than for young adults, in line with some previous findings in the literature (see, e.g., Castel, 2005; Smith et al., 1998) but not with others (see, e.g., Arbuckle et al., 1990). Importantly, it occurred in an incremental fashion, with the probability of correct recognition positively related to the amount of consistency between the stimuli and existing knowledge/experience.

The results highlight a tendency for individuals to preferentially remember material that is congruent with their existing experiences and/or to preferentially forget material incongruent with their experiences. Such an effect would cause a bias in how individuals perceive their surroundings, with a tendency for established concepts to become reinforced by experience. This mechanism may allow individuals to more rapidly and accurately perceive and categorize common stimuli. It may partially explain, for example, why high-frequency words are more rapidly and accurately identified than are low-frequency words (Grainger, 1990) and how expectations based on prior knowledge facilitate the speed and accuracy of perceiving objects (Bar, 2004).

Previous research has directly compared memory for material congruent with knowledge/experience with memory for material incongruent with knowledge/experience. It is well established that familiarity with study material can aid memory. However, the present data demonstrate that this can occur in an incremental fashion on the basis of the degree of congruency between study materials and knowledge/experience. This provides evidence for a graded mechanism in which the *amount* of congruency with knowledge is important, not just the presence or absence of congruency with knowledge. This graded effect of congruency was more evident in older adults relative to young adults, with older adults showing a significant effect but young adults only a marginal effect. To our knowledge, this study presents the first suggestion of an incremental change in age-related associative deficits. This provides important insight into Naveh-Benjamin’s (2000) associative deficit hypothesis by demonstrating that associative deficits can be manipulated at a fine-grained level.

Older adults may be more influenced by prior knowledge than young adults simply because their knowledge has been reinforced over many years of experience. This argument could be applied to studies of semantically related word pairs (Naveh-Benjamin et al., 2003) in which older adults have encountered the words together across their lifetime. However, it is difficult to apply this argument to the present findings because young and older adults would not necessarily have had different exposure to images of George Bush and Prince William. Another explanation for the pattern of data observed could be that older adults have a reduced ability to inhibit prior knowledge (e.g., age-related inhibitory deficits; Hasher & Zacks, 1988). When completing the memory test, older adults may have had difficulty inhibiting the natural tendency to respond with the name that more closely matched the test face, rather than the name that they remembered seeing with the test face. Future research should aim to clarify how schematic support differentially affects the memory of young and older adults and to investigate why it particularly benefits older adults in some paradigms but not in others.

## Footnotes

1 These numbers exclude 4 older adults and 1 young adult who failed to follow instructions and a further 2 young adults who were not familiar with the famous individuals referred to in the memory test.
